# Supplementary material for: High-performance and compact-designed flexible thermoelectric modules enabled by a reticulate carbon nanotube architecture
Source: Nat Commun. 2017 Mar 24;8:14886. doi: 10.1038/ncomms14886 (PMC5477522; doi:10.1038/ncomms14886)
Supplement: Supplementary Information — Supplementary Figures, Supplementary Tables, Supplementary Notes, and Supplementary References [file ncomms14886-s1.pdf]

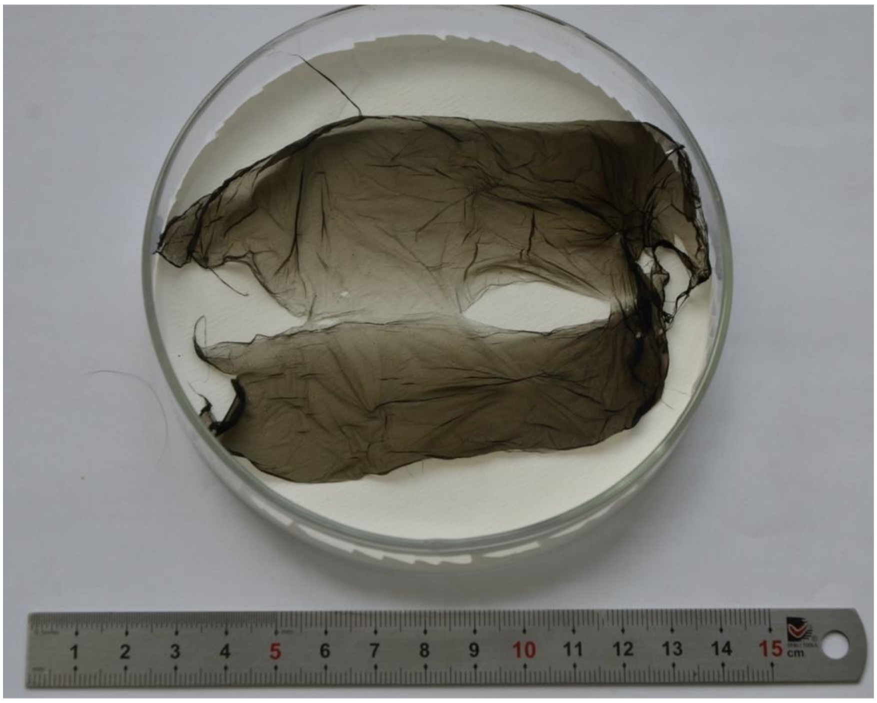

Supplementary Figure 1 | The optical photograph. The optical photograph of the as-grown SWNT film with a thickness of ~200 nm.

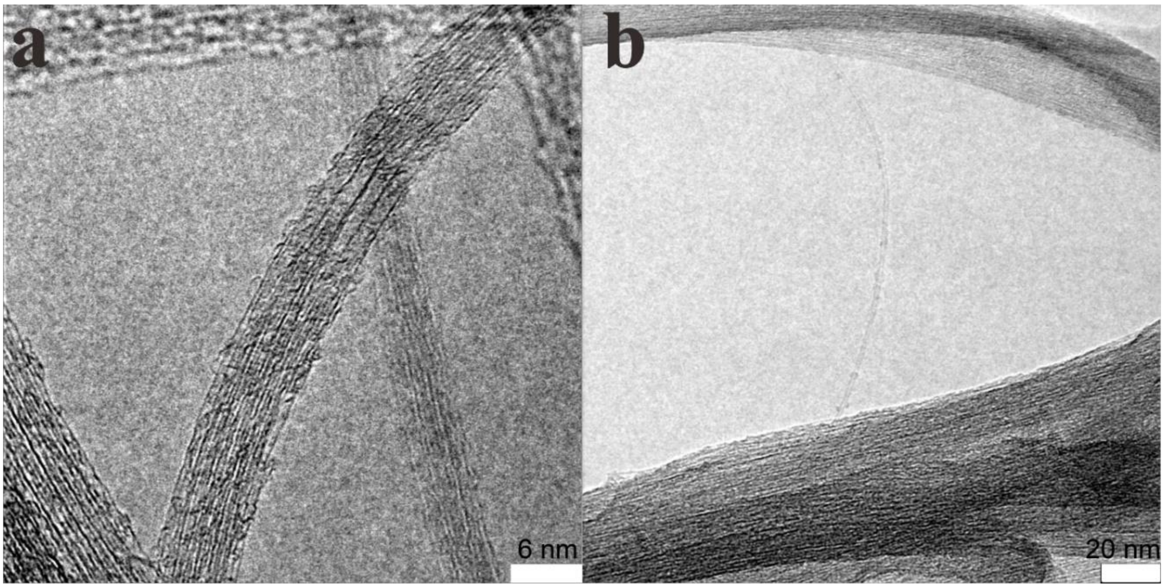

Supplementary Figure 2 | The transmission electron microscope (TEM) image. The TEM image of the as-grown CNT film. The CNTs exist mainly in the form of bundle, which are SWNTs.

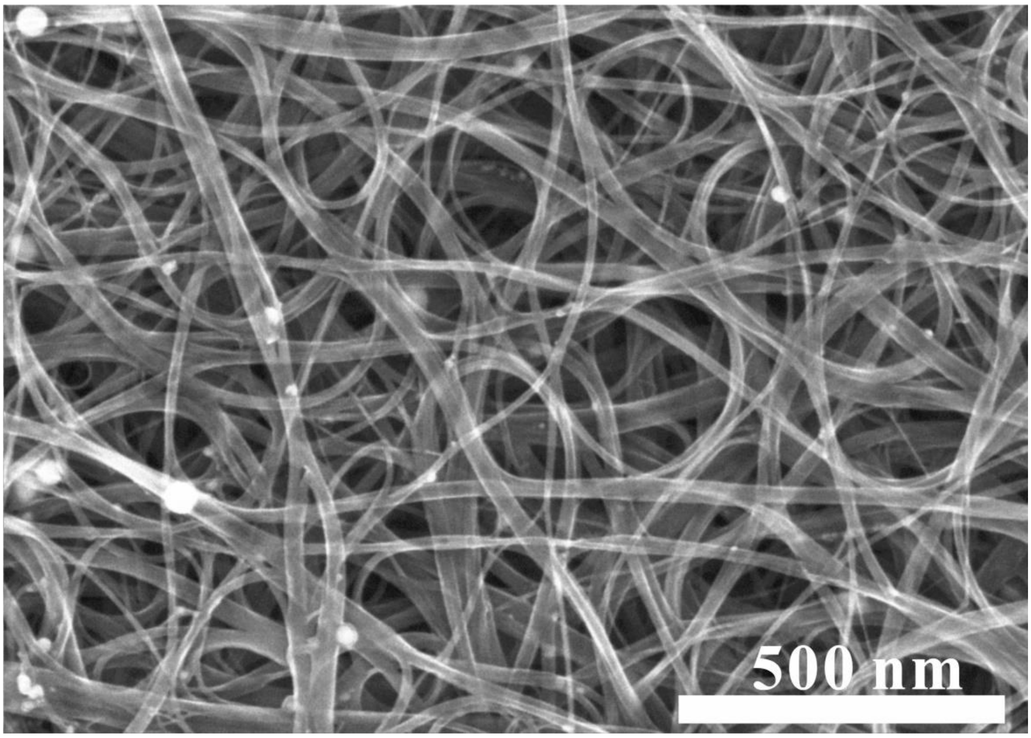

Supplementary Figure 3 | The scanning electron microscope (SEM) image. The SEM image of the as-grown SWNT film.

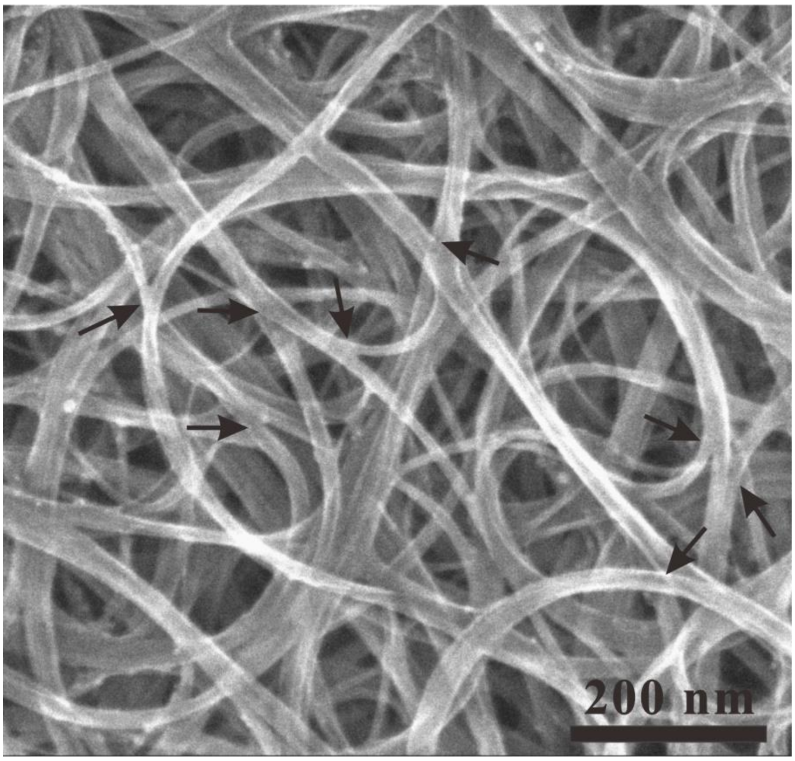

Supplementary Figure 4 | The SEM image of the as-grown CNT film at higher magnification. The black arrows in the image point out the Y-type inter-bundle junctions of hierarchical CNT continuous networks.

Supplementary Table 1. Thermoelectric properties in air at room temperature for n-type thick CNT ribbons (25 mm × 1 mm, cut from the same homogeneous CNT film with a thickness of ~3 μm) with different PEI doping concentrations (keeping the same solution volume around 30 μL)

Doping concentrations	σ S m^{-1}	S μV K^{-1}	PF μW m^{-1} K^{-2}
0.5 wt.% PEI	1.79×10^5	−52	493
1 wt.% PEI	2.32×10^5	−67	1042
2 wt.% PEI	2.56×10^5	−62	985

Supplementary Table 2. Measurement results of thermoelectric properties in air at room temperature for a CNT film with 3 μm in thickness before and after 1 wt.% PEI doing

Specimen	σ S m^{-1}	S μV K^{-1}	PF μW m^{-1} K^{-2}
Pristine CNT film	2.5×10^5	74	1369
CNT film doped with 1 wt.% PEI	2.32×10^5	−67	1042

Supplementary Note 1:

Thermal conductivity measurement

The figure of merit of thermoelectric (TE) materials is very important to evaluate the module. At present it is quite challenge to determine the accurate thermal conductivity of CNT film in our work, which requires specific and advanced measuring techniques. It is for this reason that previous researchers usually estimated the ZT by measuring out-of-plane thermal conductivity of CNT films, while the

power factor was measured in the in-plane direction. The obtain ZT is actually inaccurate because of the anisotropy in the film. Recently, researchers have carried out the measurement of in-plane thermal conductivity for thick CNT buckypaper by a self-heating method, in which non-contact infrared thermometer was used to measure temperature distribution of the heated films.[1-3] Itkis *et al.* measured thermal conductivity of single-walled carbon nanotube (SWNT) thin films by a bolometric technique, where infrared radiation was used as a heat source and the influence of blackbody radiation was limited by a quartz filter.[4]

Here, we tried to measure the in-plane thermal conductivity of CNT films using the home-made measuring apparatus through a developed self-heating method.[5] The measurements of in-plane thermal conductivity (κ) were performed by a self-heating method based on the one-dimensional heat transfer equation, where the specimen itself serves both as a heater and a temperature sensor.

In a high vacuum of ~1 × 10^{-3} Pa, the film ribbon with larger radio of length to width fixed on the four-electrode testing stage was heated by a series of direct current (I), and corresponding steady-state resistances were measured for fitting the $R^{-1}-I^2$ curve. Then we measured the $R-T$ relationship by a small current avoiding Joule heating effect. Eventually the κ was calculated by the equation $\kappa=-\frac{L}{12A_{\mathrm{sec}}}\cdot\frac{1}{R_0}\cdot\frac{b}{k}-\frac{g_{\mathrm{rad}}L^2}{12}$, where L and A_{sec} represent the suspended length and the cross section area of the specimen, k and b is the slope of $R^{-1}-I^2$ and $R-T$ curve respectively, and R_0 is the resistance of the suspended specimen under ambient

temperature. $g_{\mathrm{rad}} = \dfrac{4\varepsilon\sigma A_{\mathrm{rad}} T_0^3}{L A_{\mathrm{sec}}}$ is defined as the radiant heat loss coefficient, where σ = 5.67×10^{-8} W m^{-2} K^{-4}, ε and A_{rad} are Stefan-Boltzmann constant, emissivity and radiant area of the specimen, respectively.

After PEI doping, it is difficult to transfer the thin CNT films (hundred nanometers in thickness) from the substrate to the testing stage. Instead, we found that it is easier to transfer the relatively thick n-type CNT films without damage from the Teflon substrate possessing low surface energy. Therefore, we measured the in-plane thermal conductivity of relatively thick CNT film (thickness is ~6 μm) before and after 1 wt.% PEI doing for investigating the thermal transport characteristic. Figure S5 a–b show the measured $R^{-1} - I^2$ and $R - T$ curve of n-type CNT film doped with 1 wt.% PEI, which both exhibit ideal linear relationships and satisfactorily agrees with our theoretical deduction. The measurement results of the in-plane thermal conductivity, electrical conductivity, Seebeck coefficient and calculated PF and ZT are listed in Table S3. The relative electronic contribution to the in-plane thermal conductivity for the n-type CNT film can be estimated on the basis of the Wiedemann-Franz law ($\kappa_e = L_0 \sigma T$) with a Lorenz number L_0 = 2.44 × 10^{-8} W Ω^{-1} K^{-2} (the Lorenz number is dependent on materials and may not be applicable to our samples),[6] which indicates the ratio of the electron to phonon contribution to the thermal conductivity is about 1 to 13, which suggests that the heat transport is dominated by the phonon component in the n-type CNT film. After 1 wt.% PEI doping, the additionally introduced interfacial thermal resistances from polymer coating enhanced phonon scattering, which results in the decrease of in-plane thermal conductivity from 24 W m^{-1} K^{-1} to 18

W m^{-1} K^{-1}. The reduced thermal conductivity is beneficial to thermoelectric performance.

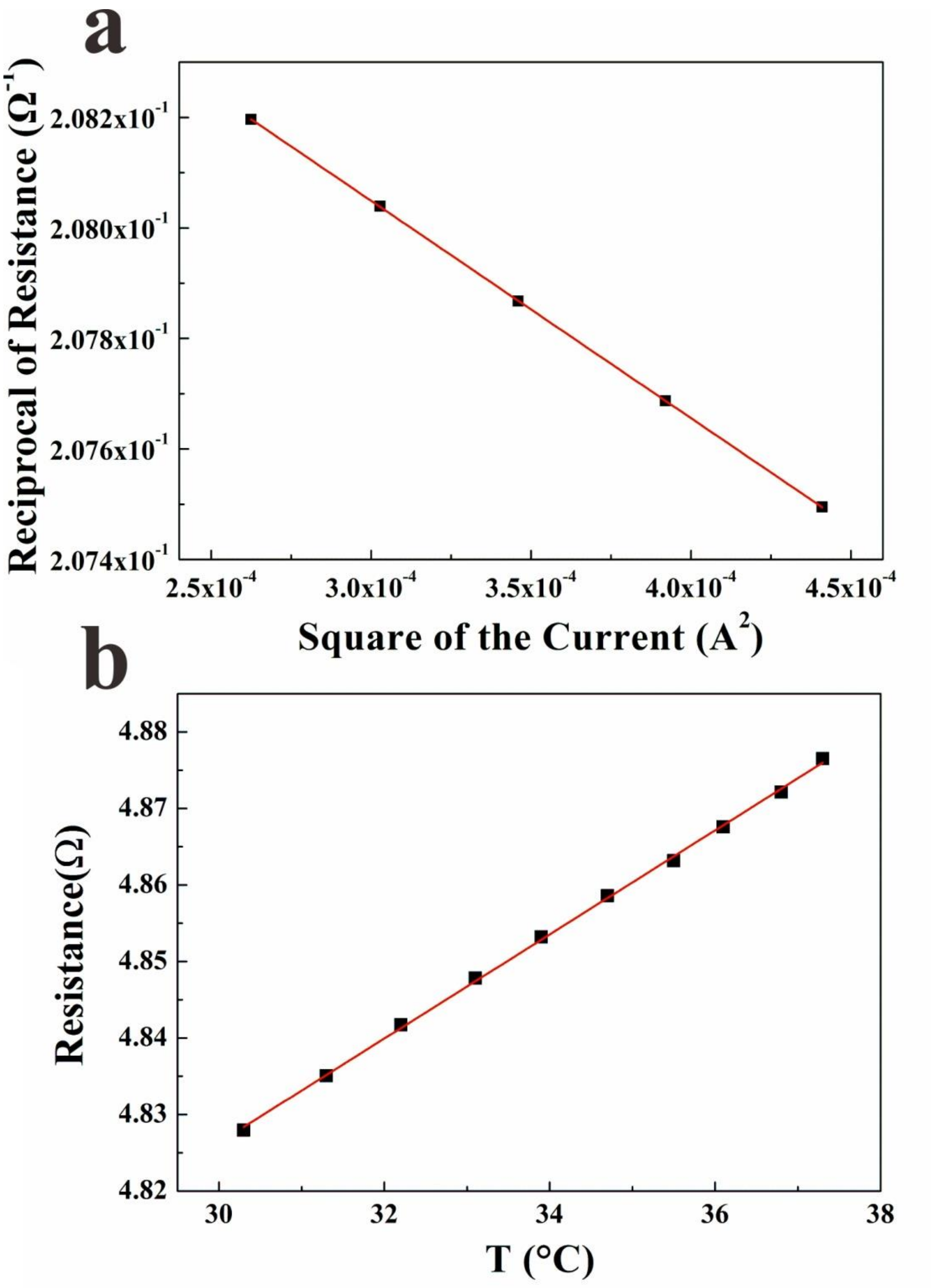

Supplementary Figure 5 | The in-plane thermal conductivity measurement. The in-plane thermal conductivity measurement of n-type CNT film doped with 1 wt.% PEI in a stabilized vacuum of 1 × 10^{-3} Pa. a) The obtained $R^{-1} – I^2$ curve. b) The measured $R–T$ curve in a small temperature range.

Supplementary Table 3. Measurement results of thermoelectric properties at room temperature for a CNT film with 6 μm in thickness before and after 1 wt.% PEI doing

Specimen	σ<br>$S\ m^{-1}$	S<br>$\mu V\ K^{-1}$	κ<br>$W\ m^{-1}\ K^{-1}$	PF<br>$\mu W\ m^{-1}\ K^{-2}$	ZT
Pristine CNT film	2.23×10^5	87	24	1691	0.021
CNT film doped with 1 wt.% PEI	1.78×10^5	−82	18	1197	0.02

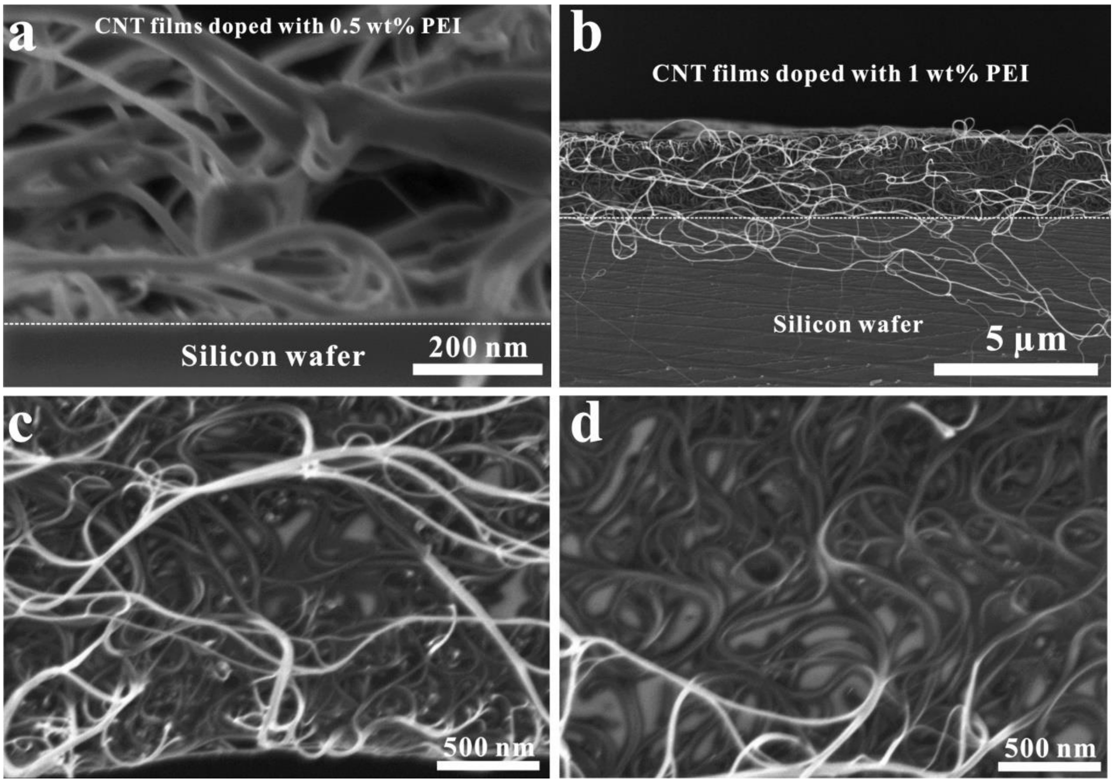

Supplementary Figure 6 | The cross-section SEM images. The cross-section SEM images of n-type CNT films deposited on silicon substrates. a) The cross-section SEM images of thin CNT films doped with 0.5 wt% PEI. b–c) The cross-section SEM images of relatively thick CNT films doped with 1 wt% PEI. It seems like that PEI penetrate in the pores of CNT network.

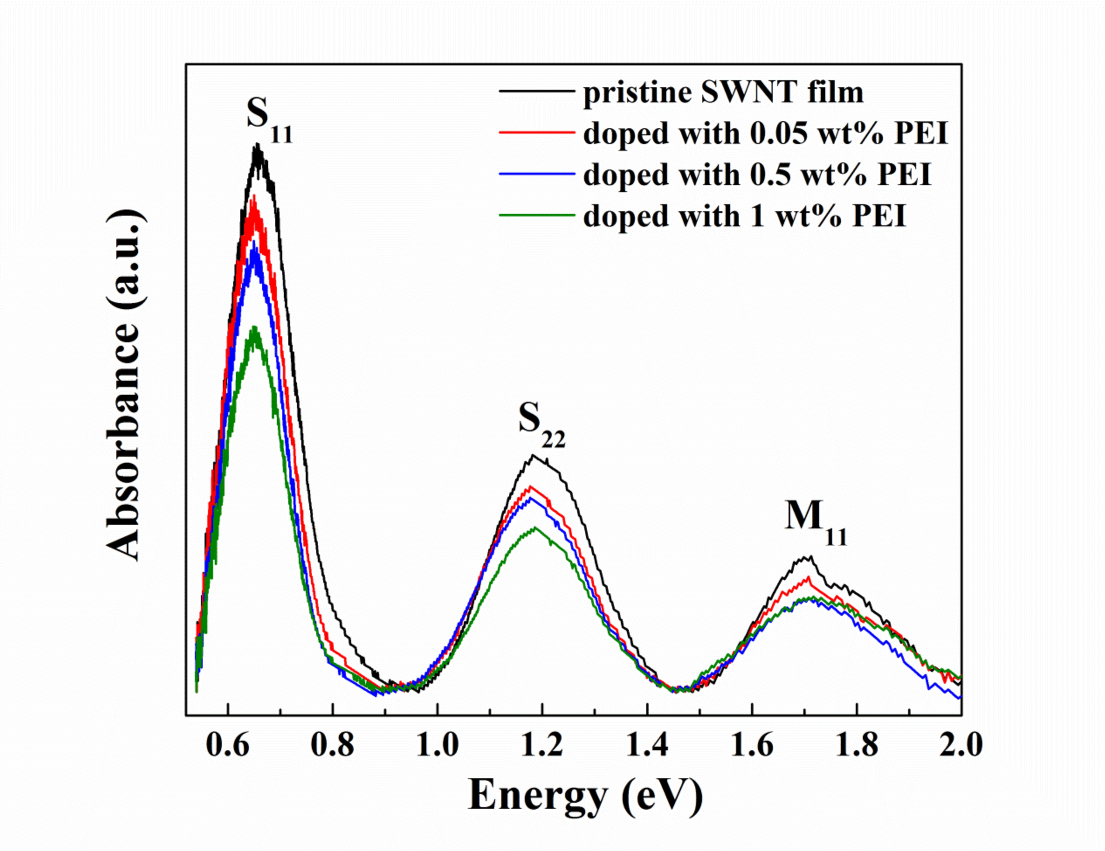

Supplementary Figure 7 | The absorption spectra. The normalized absorption spectra removed absorbing background of π electron plasmon of the pristine and doped SWNT films.

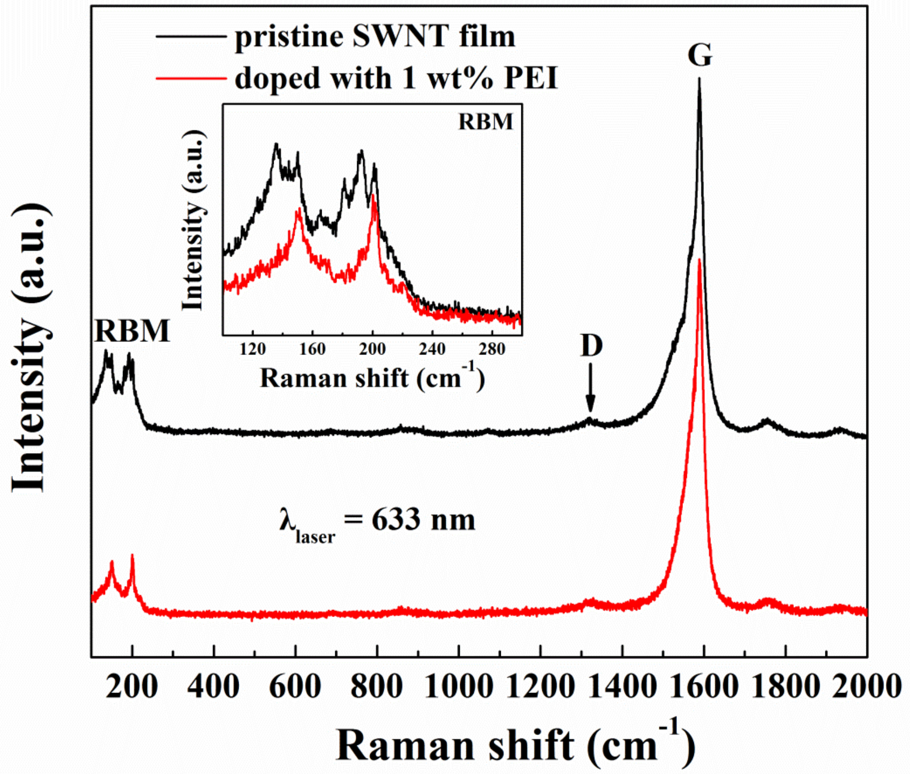

Supplementary Figure 8 | The normalized Raman spectra. The normalized Raman spectra excited with a 633 nm laser for the SWNT film before (black trace) and after (red trace) 1wt.% PEI doping.

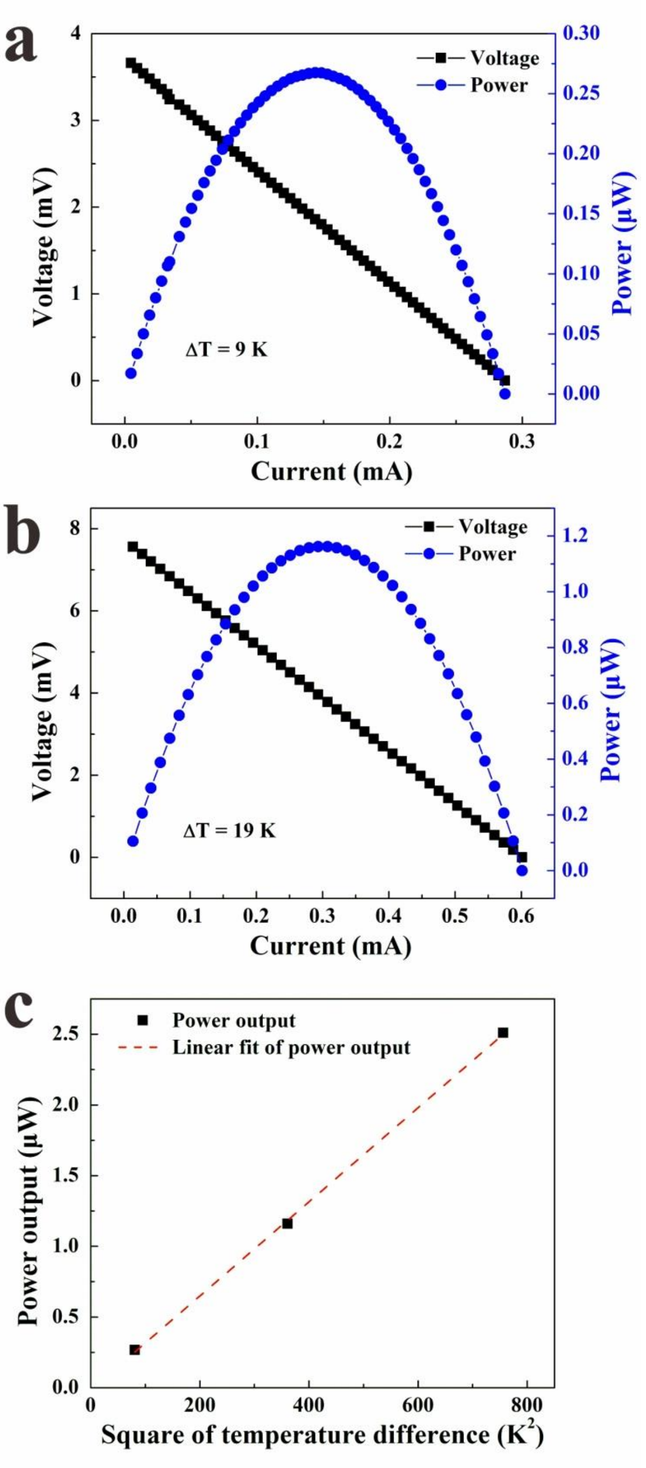

Supplementary Figure 9 | The thermoelectirc performance in different temperature differences. The voltage–current curve and power–current curve of the module at the temperature difference of a) 9 K and b) 19 K. c) The dependence of measured power out on the square of ΔT, which indicates the power out approximately increased linearly with the square of ΔT.

Supplementary References

1. Zhang, L., Zhang, G., Liu, C. H. & Fan, S. S. High-Density Carbon Nanotube Buckypapers with Superior Transport and Mechanical Properties. *Nano Lett.* **12**, 4848-4852 (2012).

2. Wang, D., Song, P. C., Liu, C. H., Wu, W. & Fan, S. S. Highly oriented carbon nanotube papers made of aligned carbon nanotubes. *Nanotechnology* **19**, 075609 (2008).

3. Zhang, G., Liu, C. H. & Fan, S. S. Directly measuring of thermal pulse transfer in one-dimensional highly aligned carbon nanotubes. *Scientific Reports* **3**, 2549 (2013).

4. Itkis, M. E., Borondics, F., Yu, A. P. & Haddon, R. C. Thermal conductivity measurements of semitransparent single-walled carbon nanotube films by a bolometric technique. *Nano Lett.* **7**, 900-904 (2007).

5. Zhou, W. B. *et al.* Ultrahigh-Power-Factor Carbon Nanotubes and an Ingenious Strategy for Thermoelectric Performance Evaluation. *Small* **12**, 3407-3414 (2016).

6. Yu, C., Choi, K., Yin, L. & Grunlan, J. C. Light-Weight Flexible Carbon Nanotube Based Organic Composites with Large Thermoelectric Power Factors. *Acs Nano* **5**, 7885-7892 (2011).
